# *TP53* Mutations as a Driver of Metastasis Signaling in Advanced Cancer Patients

**DOI:** 10.3390/cancers13040597

**Published:** 2021-02-03

**Authors:** Ritu Pandey, Nathan Johnson, Laurence Cooke, Benny Johnson, Yuliang Chen, Manjari Pandey, Jason Chandler, Daruka Mahadevan

**Affiliations:** 1 Cancer Center, University of Arizona, Tucson, AZ 85724, USA; lcookeaz@gmail.com (L.C.); yuliangchen@email.arizona.edu (Y.C.); 2 Department of Cellular and Molecular Medicine, University of Arizona, Tucson, AZ 85724, USA; 3 School of Medicine, Vanderbilt University, Nashville, TN 37325, USA; nathan.r.johnson@vanderbilt.edu; 4 MD Anderson Cancer Center, Houston, TX 77030, USA; bjohnson6@mdanderson.org; 5 West Cancer Center, 7945 Wolf River Blvd, Germantown, TN 38138, USA; mpandey@westclinic.com (M.P.); jachandler@westclinic.com (J.C.); 6 Mays Cancer Center, University of Texas Health San Antonio, San Antonio, TX 78229, USA

**Keywords:** NGS, FISH, IHC, oncogenes, tumor suppressors, *TP53*, targeted therapy

## Abstract

**Simple Summary:**

The DNA sequencing of cancer provides information about specific genetic changes that could help with treatment decisions. Tumors from different tissues change over time and acquire new genetic changes particularly with treatment. Our study analyzed the gene changes of 171 advanced cancer patients. We predicted that tumors gain new mutations but that TP53 mutations (guardian of the human genome) are conserved as the tumor progresses from primary to metastatic sites and across tissue types. We analyzed the primary and metastatic site gene changes in 25 tissue types and conducted in-depth analysis of colon and lung cancer sites for substantial changes. TP53 site specific mutations were different across tissue types and suggest different molecular changes. Other genetic changes that occur together with TP53 as drivers collectively alter how cells respond to signals which are important to tumor treatments.

**Abstract:**

Molecular profiling with next generation sequencing (NGS) delivers key information on mutant gene sequences, copy number alterations, gene-fusions, and with immunohistochemistry (IHC), is a valuable tool in clinical decision making for patients entering investigational agent trials. Our objective was to elucidate mutational profiles from primary versus metastatic sites from advanced cancer patients to guide rational therapy. All phase I patients (*n* = 203) with advanced cancer were profiled by commercially available NGS platforms. The samples were annotated by histology, primary and metastatic site, biopsy site, gene mutations, mutation count/gene, and mutant *TP53*. A molecular profile of each patient was categorized into common and unique mutations, signaling pathways for each profile and *TP53* mutations mapped to 3D-structure of p53 bound to DNA and pre/post therapy molecular response. Of the 171 patients analyzed, 145 had genetic alterations from primary and metastatic sites. The predominant histology was adenocarcinoma followed by squamous cell carcinoma, carcinoma of unknown primary site (CUPS), and melanoma. Of 790 unique mutations, *TP53* is the most common followed by *APC*, *KRAS*, *PIK3CA*, *ATM*, *PTEN*, *NOTCH1*, *BRCA2*, *BRAF*, *KMT2D*, *LRP1B*, and *CDKN2A. TP53* was found in most metastatic sites and appears to be a key driver of acquired drug resistance. We highlight examples of acquired mutational profiles pre-/post- targeted therapy in multiple tumor types with a menu of potential targeted agents. Conclusion: The mutational profiling of primary and metastatic lesions in cancer patients provides an opportunity to identify *TP53* driver ‘pathways’ that may predict for drug sensitivity/resistance and guide rational drug combinations in clinical trials.

## 1. Introduction

‘Precision oncology’ or ‘cancer genome medicine’ is the seamless application of the Watson–Crick ‘central dogma’ to every patient with cancer (personalized therapy), where their tumor molecular profile (genome, proteome, epigenome, immunome, micro-environmentome, metabolome, etc.) may inform diagnosis, prognosis, and treatments. ‘Precision therapeutics’ of cancer implies mechanism of action-based targeting of the ‘hallmarks’ of cancer utilizing molecular taxonomy, genomic, proteomic in diagnosis and guiding therapeutics in well-designed innovative trials. The large-scale whole genome sequencing (WGS) of cancer such as the ICGC (International Cancer Genome Consortium) and TCGA (The Cancer Genome Atlas) have cataloged prevalent genomic alterations across a myriad of human malignancies [1,2], identifying recurrent genetic mutations that drive aberrant signaling pathways controlled by master regulators that lead to acquired targetable phenotypic characteristics [3]. With the advent of new molecular and cellular technologies, oncology has evolved from treating cancer patients with non-specific DNA-damaging and microtubule-targeted combination chemotherapy to molecular pathology-stratified histology agnostic immune checkpoint and targeted therapies.

Intra-tumor and spatially separated multiple sub-clone heterogeneity elegantly described in clear cell renal cancer and other malignancies are major contributors to the understanding of tumor evolution and drug resistance. Branched ‘Darwinian’ evolution [4] is a significant challenge to current therapies but should provide insights to mitigate or disrupt anticipated genetic alterations by a rationalized approach to precision therapeutics. Elucidating the mechanism of genomic alterations is likely to identify master regulators [5] once longitudinal tumor sampling with minimally invasive methods become a reality. Solid and liquid tumor biopsies during the course of therapy of a given cancer subtype are likely to inform decisions to switch tailored therapies that make mechanistic sense (e.g., TRACERx-Tracking Cancer Evolution through Therapy [6] in lung cancer [7]) and advanced cancer [8]. The current practice of stratifying patients to a single-diagnostic/single-drug will change as multiple biomarkers has become clinically actionable. The development of multi-biomarker assays coupled to NGS (DNA and RNA sequencing and single cell transcriptomics) will complement ‘precision’ diagnostics, prognostics, and therapeutics [9].

A major requirement of ‘precision oncology’ is hypothesis-driven research to improve patient outcomes [10]. NGS data have been utilized to conduct histology-agnostic ‘umbrella’ or ‘basket’ trials and one such trial sponsored by the NCI (MATCH trial) [11] where multiple histologies are matched by biomarkers to a targeted agent. A second approach uses a master protocol (S1400) within a single histology (e.g., squamous cell lung cancer) to test multiple omic-drug matches based on a defined set of genes. A third approach is a ‘strategy’ trial where patients are assigned a therapy based on their omics profile or physician’s choice. The NCI has launched the ‘The Molecular Profiling-based Assignment of Cancer Therapeutics’ (M-PACT) [12] trial where patients are screened for actionable gene alterations and randomized to a drug that targets a mutated oncogene product or a drug chosen by a physician that does not correspond to a mutation or amplification. The primary endpoint is response rate and four-month progression-free survival. A fourth approach is an ‘observational’ trial utilizing off-label targeted therapies (Targeted Agent and Profiling Utilization Registry–TAPUR) [13]. A major challenge is analysis of large-scale genomic data for clinical application [14]. Whole-genome sequencing is an excellent strategy for comprehensive molecular profiling but requires validation for clinical utility. The currently available gene-capture platforms, when used to match therapies with whole exome, whole genome, and transcriptome (RNA-Seq), are in the domain of discovery research [15].

In unmatched and matched paired analysis of primary and metastatic tumors, *TP53* appears to enrich to metastasis [16]. However, whether the cause and/or effect is due to chromosomal instability and/or drug resistance remains to be established [16]. In our ‘New Therapeutics Program’ all advanced cancer patients were molecularly profiled by NGS for mutations, copy number alterations (CNA), translocations (FISH/CISH), and IHC to inform clinical decisions for investigational agent trials. We hypothesized that for primary and metastatic sites, *TP53* mutations are more frequent and appear to be a key driver of acquired drug resistance in advanced cancer patients. Mutation in *TP53* is heterogeneous and are known to induce complex transcriptional changes effecting multiple biological responses [17]. Cellular responses to a specific TP53 mutation may depend on the tissue type, tumor stage and co-mutated genes. Thus, investigating the underlying p53 driven signaling pathways is predicted to provide opportunities to not only understand evolving pathobiology but also provide better guidance to rational drug therapies.

## 2. Results

### 2.1. Primary and Metastatic NGS Profiling of Advanced Cancer Patients

Of the 171 patients entering phase I clinical trials, 145 had genetic alterations with respect to the primary and metastatic site, histology, and total number of clinically relevant mutated genes (Table 1). In our cohort of patients, utilizing large NGS platforms probing 300 (Foundation One: 25% Patients, 421 (Precipio: 36% Patients), and 600 (Caris MI: 39% Patients) cancer-related genes identified (a) common tumor types with a myriad of total unique mutations e.g., non-small cell lung cancer 85 mutations (*n* = 20), colon cancer 87 mutations (*n* = 28), breast cancer 79 mutations (*n* = 17), and ovarian cancer 51 mutations (*n* = 14); (b) common tumor types with a lower average number of uniquely mutated genes per patient e.g., non-small cell lung cancer (*n* = 3), colon cancer (*n* = 3), breast cancer (*n* = 4), and ovarian cancer (*n* = 3); (c) some tumor types have a higher number of unique mutated genes per patient e.g., anus (*n* = 27 mutations), CUPS (*n* = 90 mutations), esophagus (*n* = 54), prostate (*n* = 49 mutations) and uterus (*n* = 38 mutations); (d) common driver mutations per histologic type were for e.g., for colon cancer (*TP53*, *APC*, *KRAS*, *PIK3CA*, *BRAF*, *SMAD4*, *SPTA1*), non-small cell lung cancer (*TP53*, *EGFR*, *KRAS*, *PTEN*, *CDKN2A*, *NOTCH1*, *NTRK1*), breast cancer (*TP53*, *NOTCH1*, *PIK3CA*, *ARID1A*, *BRCA2*, *NF1*, *ABL1*) and ovarian cancer (*TP53*, *FLT4*, *ALK*, *ATM*, *BRAF*, *BRIP1*, *C11orf30*) respectively.

**Figure 1:**
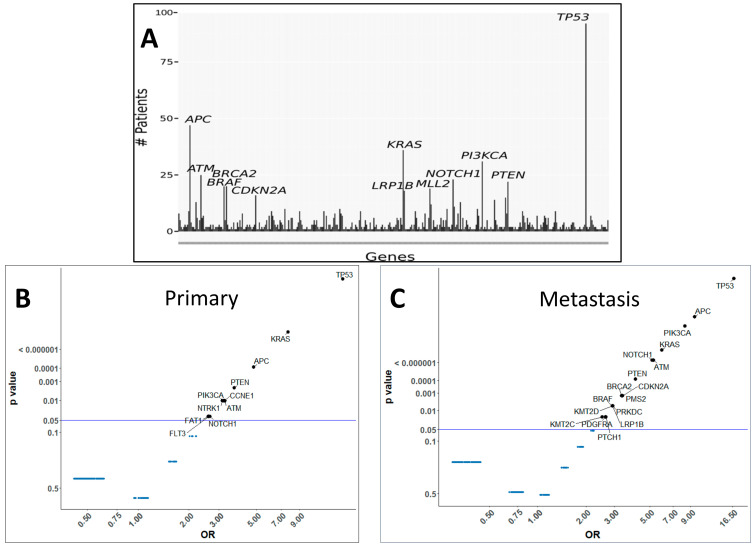
The mutation frequency of the common drivers of malignancy. (**A**) Most frequently mutated genes for 171 advanced cancer patients in study. Top 12 mutated genes labeled with gene name. Plot showing representation of gene mutations with significance (*p*-values < 0.05) across (**B**) primary samples and (**C**) metastatic samples. Y-axis is *p*-value and X-axis is the odds ratio. Significant genes are labeled and highlighted.

Of the 171 phase I cancer patients with mutations, adenocarcinoma is the most common (*n* = 111), followed by squamous cell carcinoma (*n* = 17), CUPS (*n* = 11) and melanoma (*n* = 6). We determined the average number of unique mutations per patient for adenocarcinoma (*n* = 7), squamous cell carcinoma (*n* = 6), CUPS (*n* = 10), and melanoma (*n* = 8). In our patient cohort, mutation frequency among the drivers of malignancy, *TP53* had the highest frequency (92, ~54%), followed by *APC* (47, ~27%), *KRAS* (36, ~27%), *PIK3CA* (31, ~18%), *ATM* (25, ~15%), *NOTCH1* (23, ~7%), *PTEN* (22, ~13%), *BRCA2* (20, 12%), *BRAF* (20, ~12%), *KMT2D* (19, ~11%), *LRP1B* (18, ~11%) and *CDKN2A* (16, ~9%) (Figure 1A). Our study identified gene mutations unique to primary and metastatic sites that track with common mutations. Individual patient data was further screened for accurate and unique identification of primary or metastatic biopsy sites resulting in 145 patient samples. The frequency of individual gene mutations in primary and metastatic samples of 145 patients across all tumor types was estimated. Some of the gene mutations were found to be prevalent with significance to both primary and metastatic tissues, while others occurred with higher frequency in primary or metastatic sites (Figure 1B,C).

**Figure 2:**
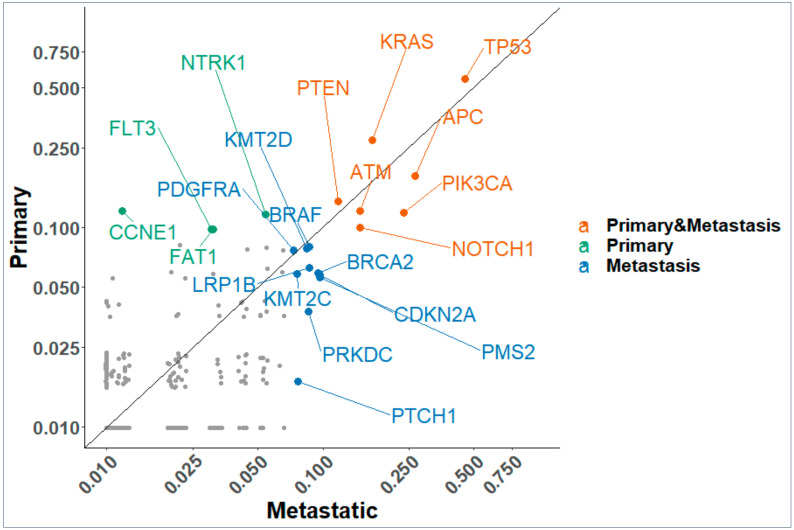
Scatter plot of frequencies of gene mutations in primary and metastatic samples. Genes that are enriched in primary, metastatic or both samples with *p* value < 0.05 are labeled.

The relative frequency of *TP53* mutations amongst other genes was 0.54 in primary (*p* value (val) = 7.9 × 10^−28^) and 0.45 (*p* val = 1.42 × 10^−41^) in metastatic tissue samples (Figure 2) respectively. *TP53* was the most frequent gene in both primary and metastatic sites followed by *KRAS*, *APC*, *PTEN*, *PIK3CA*, *ATM*, and *NOTCH1*. In addition, Figure 2 shows that metastatic sites carry several new mutations with higher frequency (*KMT2D*, *BRAF*, *BRCA2*, *KMT2C*, *PRKDC*) along with the top driver mutations found in primary sites. There is a combination of cell signaling genes, DNA damage repair, and histone methyltransferase genes that are common to primary and metastatic sites and some unique to metastatic sites. The enrichment of multiple DNA damage repair proteins in metastatic sites suggest potential for investigating the safety and efficacy of PARP inhibitors therapy in a ‘basket’ trial. It is well known that there is an interplay between epigenetic pathways and *TP53* mutations. We see a statistically significant enrichment of mutations of the *KMT2* family of histone modifying genes. These proteins are part of multimeric complexes that bind with other proteins to target enhancers across the genome that impact complex gene regulation. Their mutated frequencies in tumors have been reported [18] and are critical co-occurring mutations which opens possibilities for pharmacologic intervention that target cofactors in gene regulation complexes.

### 2.2. Gene Mutation Networks in Lung and Colon Cancer

**Figure 3:**
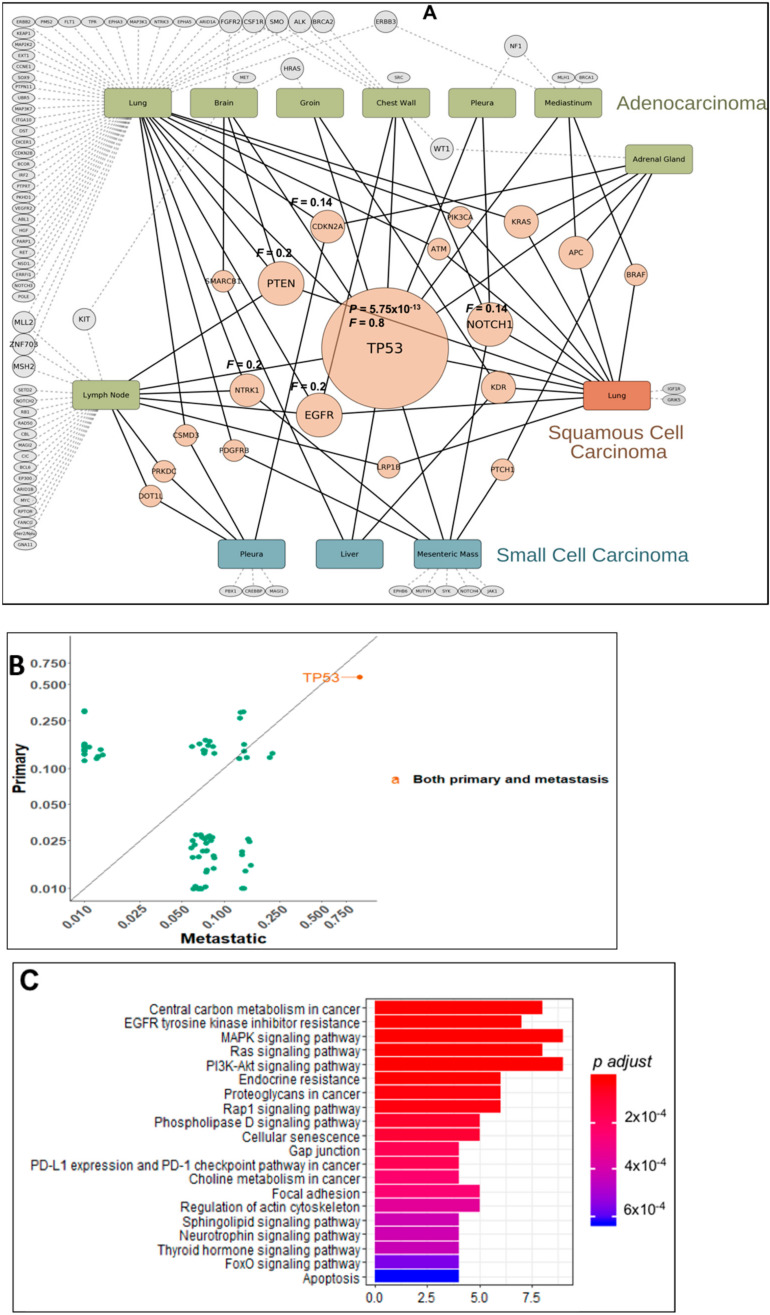
(**A**) Map of mutation frequencies in lung cancer patients. “Gene” node sizes positively correlate with total mutation frequency (F) of the gene among all primary/metastatic sites. Rectangular nodes: Primary/metastatic sites colored by histology type. Pink-colored gene nodes: Genes mutated in multiple primary/histology types. Gray-colored genes nodes: Mutated genes unique to a particular site. (**B**) Plot of relative frequency of gene mutations in primary and metastatic and gene enriched are highlighted. (**C**) Signaling pathways altered by significant gene mutation. Genes with significant mutations were analyzed for enrichment of Kegg pathways. Pathways with adjusted *p* value < 0.05 were selected. The plot shows the significant pathways with bar height as gene counts.

The system-wide profiling of pathogenic mutations in human cancer produces lists of genes that can be evaluated for their collective functions in order to garner new knowledge. Well annotated lists of genes can be input for enrichment into existing lists from prior knowledge. This methodology was applied to the overrepresented gene mutations across all primary and metastatic sites in lung and colon cancer cohorts of patients. Lung cancer patients were divided into adenocarcinoma, squamous cell carcinoma, and small cell lung cancer for this analysis. Figure 3A shows the site of biopsy for each subtype of lung cancer with their mutational profile, frequency and significant enrichments of genes. Node sizes reflect the abundance of a particular gene mutation. Although metastatic tumors harbor an increased number of genetic alterations, some of the alterations found in the primary tumor are preserved. Cytoscape analysis demonstrate that *TP53* mutations are a major central node with combined Frequency (F) of 0.8 in primary and metastatic tumors, dictating the 3 subtypes of lung cancer pathogenesis. Statistical analysis shows *TP53* enrichment with significance in both primary (*p* value = 0.01) and metastatic sites but a higher frequency and significance in metastatic patients (*p* value = 5.75 × 10^−13^) (Figure 3B). For adenocarcinoma of the lung, the *TP53* oncogenic program impacts genetic aberrancies in all the hallmarks of cancer which includes alterations in the cell cycle, DNA repair, epigenetic regulation, growth factor receptor RAS-MAPK signaling, GPCR signaling, apoptosis, and stemness pathways. We evaluated the top mutated genes (Figure 3A) for significant enrichment of Kegg pathways, Figure 3C shows the altered signaling pathways based on the top mutations and many of the pathways known for alteration in TP53 mutated tumors are prevalent. For squamous cell carcinoma of the lung, the mutational burden is less than that for adenocarcinoma, however, *TP53* mutations are a driver and associate with several known oncogenes (*KRAS*, *BRAF*, and *PI3KCA*). For small cell lung cancer, *TP53* mutations associate with angiogenic factors (*KDR*, *PDGFRB*, *EPHB6*), stem cell markers (*NOTCH1*, *4* and *PTCH1*), and a novel *NTRK1* mutation.

**Figure 4:**
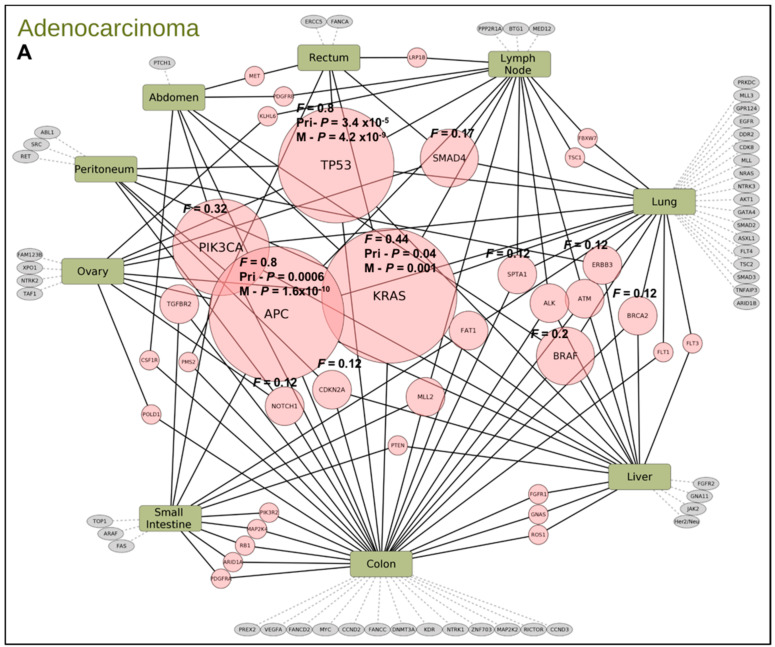

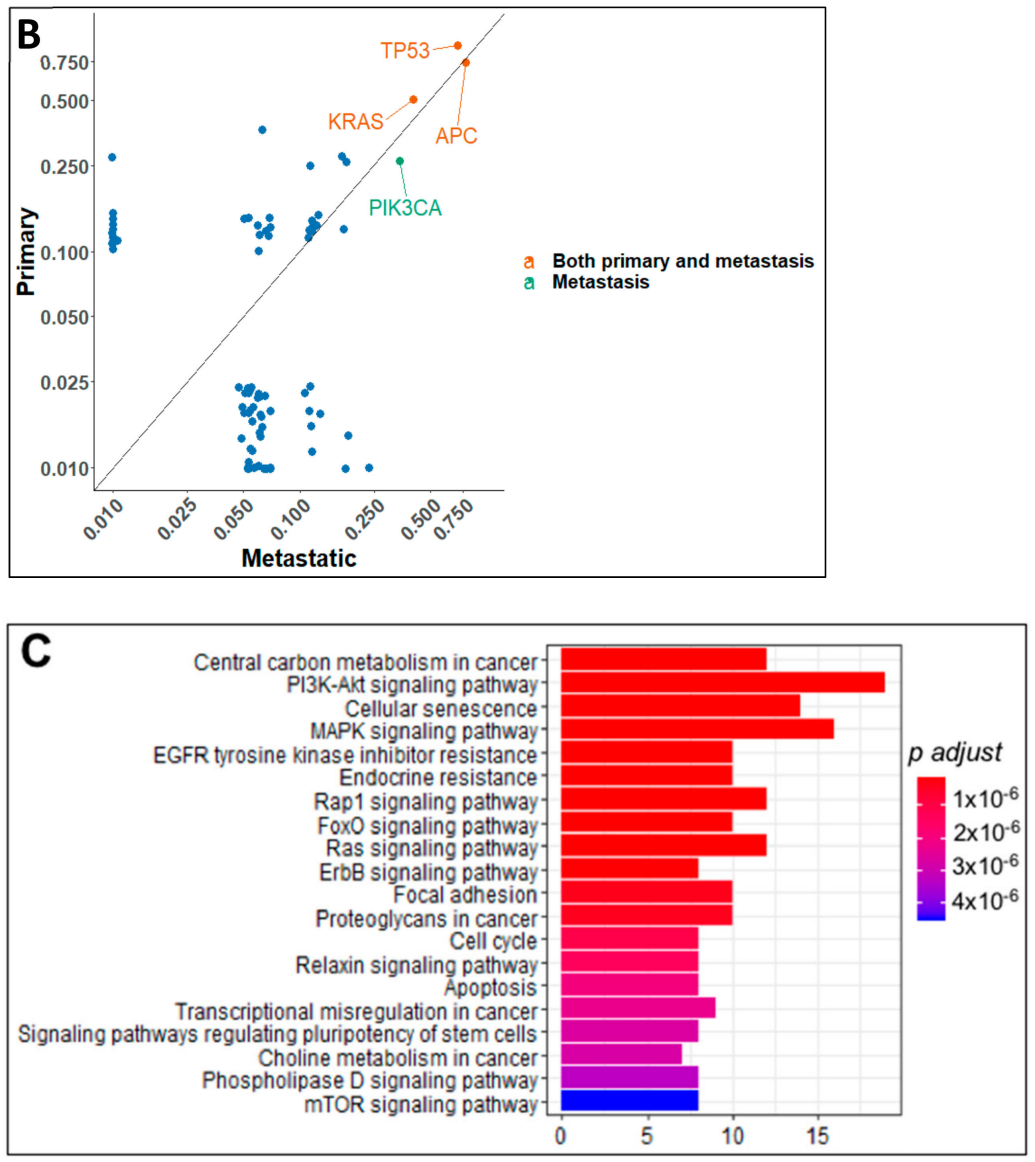
(**A**) Map of mutation frequencies (F) in colon cancer patients with adenocarcinoma histology type. “Gene” node sizes positively correlate with gene mutation frequency. Pink-colored gene nodes: genes mutated in multiple primary/metastatic sites. Gray-colored gene nodes: mutated genes unique to the particular primary/metastatic site. (**B**) Plot of relative frequency of gene mutations in primary and metastatic and gene enriched are highlighted. (**C**) Signaling pathways altered by significant gene mutation. Genes with significant mutations were analyzed for enrichment of Kegg pathways. Pathways with adjusted *p* value < 0.05 were selected. The bar plot shows the significant pathways with bar height as gene counts.

For colon cancer, statistical analysis showed the primary site mutations with combined frequency in primary and metastatic sites of 0.8 in *APC* and *TP53* that are the major drivers followed by *KRAS* (*F* = 0.4), *PIK3CA* (*F* = 0.32), *BRAF* (*F* = 0.2) and *SMAD4* (*F* = 0.17) pathogenic mutations respectively (Figure 4A). *TP53* mutations are higher in metastatic sites but new co-mutations become prevalent in metastatic colon tumor. Statistical analysis shows *TP53* enrichment with significance occurs in both primary (*p* value = 4.2 × 10^−9^) and metastatic sites (*p* value = 3.5 × 10^−5^). In a study [19] that evaluated p53 mutations in primary and metastatic tumors and CTCs from colorectal cancer (CRC) patients reported identical *TP53* mutations in both sites. However, in another study *TP53* mutations were shown to enrich to metastatic sites [16]. Of the metastatic sites, lung cancer shows the most diverse profile compared to colon. All other metastatic sites have only a few unique mutations (Figure 4A). As known from previous studies, the top 4 driver mutations in our colon cancer cohort with statistical significance are *TP53*, *KRAS,* and *APC* with *PIK3CA* (Figure 4B) mutations that are more prevalent in metastatic sites as these are late occurring mutations in CRC. Figure 4C shows signal transduction pathways affected by significant mutations in colon cancer (Figure 4A). Signaling pathway changes known in *TP53* mutated tumors are overrepresented in the list of pathways. A *RAS* wild type colon cancer patient with HER2+ by CISH and IHC 3+ had 2 pathogenic *TP53* mutations affecting both alleles indicating a rare and novel event. Similar profiles have also been generated for breast cancer (ER/PR, HER2/Neu, TNBC, TPBC), ovarian cancer, and CUPS. Data provides information for potential targeted functional studies.

### 2.3. Distribution of TP53 Mutations among Tumor Types

In our cohort of patients, *TP53* is mutated more often in lung (80%), colon = pancreas (75%), CUPS (63%), and breast (41%) cancers. No *TP53* mutations were detected in the rectum, anus, appendix and melanoma (Table 2). The type of *TP53* mutation is variable with most affecting the central DNA-binding core domain and to a lesser degree the C-terminal domain that down-regulates DNA binding to the central domain and the acidic N-terminus transcription-activation domain. We hypothesized that distinct *TP53* mutations may track with unique co-mutations that orchestrate distinct transcriptional programs and signaling pathways.

**Figure 5:**
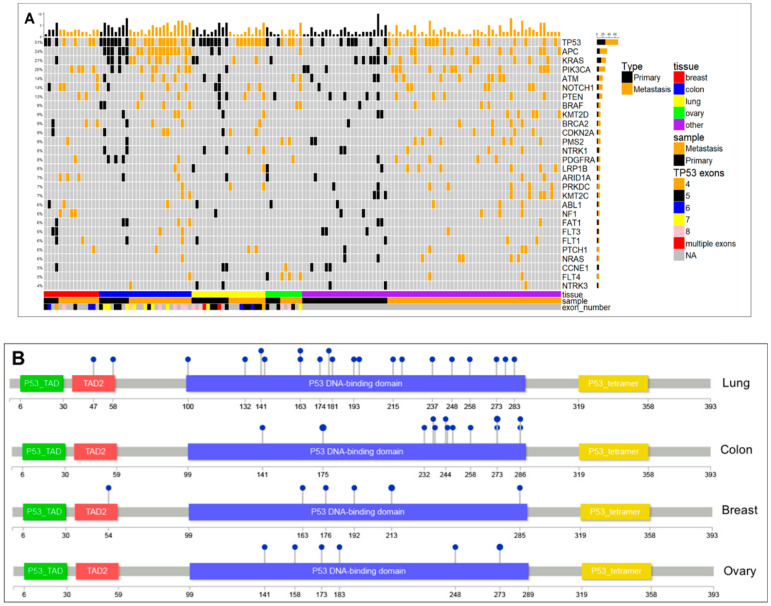
(**A**) Mutational profile of all patients from all tumor types. The figure shows frequency of mutations and differences in primary and metastatic tissue for different tumor types. (**B**) Schematic diagram of *TP53* gene and its site-specific mutations in lung, colon, breast, and ovarian tumor tissues.

We evaluated *TP53* in the context of other oncogenes that track with it at metastatic sites in each tumor site (Figure 5A). Some *TP53* mutations are conserved in both primary and metastatic sites, while new mutations are acquired in metastatic samples. Loss of p53 function via missense or truncating mutations occurs in many human tumors. Over 75% of *TP53* mutations result in the loss of wild-type function which exerts dominant-negative regulatory effects over co-expressed wild-type p53. Mutant p53 may be oncogenic in ways not related to those associated with wild type p53 functions [20,21] which include cell invasion, migration, scattering, survival, proliferation, angiogenesis, stem cell expansion, and tissue remodeling. We characterized individual *TP53* mutations in each tumor type to better understand the diversity of mutant sites (Figure 5B).

In lung cancer, *TP53* mutations are spread across the DNA binding domain (DBD). In the colon, *TP53* mutations are clustered toward the c-terminal end of the DBD which directly interacts with DNA (Figure S1). For breast and ovarian cancer, *TP53* mutations are clustered more in the middle of the DBD (Figure 5B). It is known that small changes in p53 protein do not necessarily preclude expression with some wild-type activities. Analyses of *TP53* mutation-site clustering indicate that DNA-binding activity direct versus indirect, may alter the target gene transcription affecting p53-dependent signaling pathways.

*TP53* mutations are divided into 2 categories: structural mutants, where protein folding is altered and DNA-contact mutants, where changes in critical amino acids affect DNA binding. Well characterized structural mutants such as R175H are highly unfolded under physiological conditions. Contact mutants such as R248Q exhibit decreased structural stability compared to wild type p53 [22]. We cataloged common and unique *TP53* mutations for lung cancer (Table 3, Figure 5B) and colon cancer (Table 4, Figure 5B) with respect to aberrations documented from functional studies. In lung adenocarcinoma, mutations are observed in exon 4 (*n* = 3), exon 5 (*n* = 8), exon 6 (*n* = 4), exon 7 (*n* = 6), and exon 8 (*n* = 3). In colon adenocarcinoma, mutations occur in exon 5 (*n* = 2), exon 6 (*n* = 1), exon 7 (*n* = 8) and exon 8 (*n* = 6). Mutations in exon 4 affect transactivation while mutations in exon 5, 6 and 7 are buried within the p53 structure and affect DNA binding. Mutation in exon 5 (lung cancer) and two mutations in exon 7 (colon cancer), affect zinc binding while exon 8 mutations partially expose DNA binding.

Tetramers of p53 bind to DNA targets through two decameric half-sites separated by a variable nucleotide spacer. The spacer length (contiguous versus non-contiguous) determines affinity of protein–protein and protein-DNA interactions [20]. A crystal structure of p53 bound to DNA (Watson–Crick and Hoogsteen) was utilized to map various p53 DNA-binding domain mutations detected in our cohort of patients (Figure S1). For example, R248W (lung cancer) or R248Q (colon cancer) interfere with DNA binding affinity. H179L (lung cancer) and C238S (colon cancer) disrupt zinc binding and stabilization of a loop-sheet-helix motif necessary for protein–protein interactions. *TP53* mutations away from DNA and protein–protein interaction sites enhance structural disruption of the p53 protein that dysregulate affinity of binding to DNA targets, thus potentially driving different oncogenic signaling pathways. In addition, *TP53* mutations may be truncal or acquired at metastatic sites.

Protein–protein interaction data from Stringdb show that the p53 protein strongly interacts with multiple proteins (Figure S2A). Different mutated *TP53* sites may affect binding and interactions and in turn dysregulate p53-dependent signaling pathways. We searched for protein-interaction networks for top key mutated genes in lung and colon metastatic samples (Figure S2B,C) which showed two different networks for colon and lung with *TP53* as one of the central nodes. Some of the nodes are preserved between the two tissue types, but additional nodes produce new interactions and possible changes to integrated signaling mechanisms.

### 2.4. Pre- and Post-Targeted Therapy Response

Finally, we report on the molecular profiles pre- and/or post-treatment of 10 advanced cancer patients undergoing targeted (small molecule and monoclonal antibodies) and immune checkpoint therapy to highlight unique pathways with insights to molecular responses (Table 5). The most intriguing is the effect of targeted therapies on epistasis, a phenomenon where the effect of one gene depends upon the genetic background or presence of other modifier genes. Moreover, in contrast to individual mutations, combinations of epistatic mutations may have unique effects including unexpected phenotypes and inherent resistance to single agent therapies.

**Figure 6:**
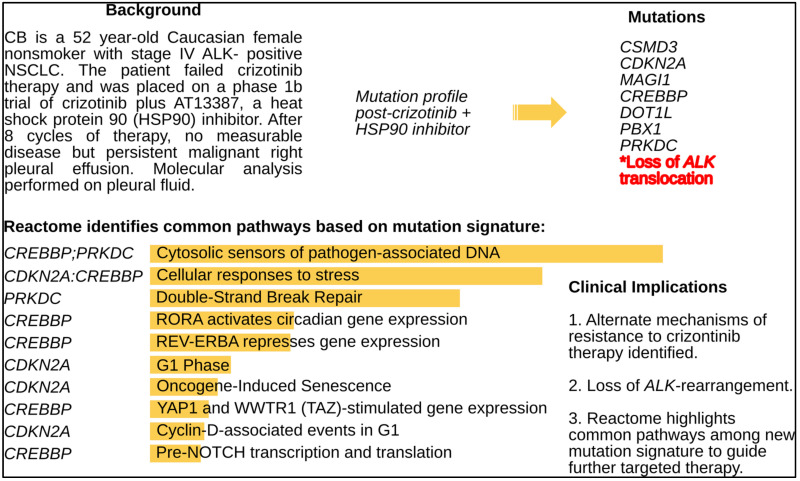
Targetable signaling pathways with of loss EML4-ALK in a patient (Case #9) with non-small cell adenocarcinoma of the lung cancer (Case #9) progressing on crizotinib plus an HSP90 inhibitor.

Case #9 is of a patient with an EML4-ALK inversion non-small cell adenocarcinoma of the lung treated with crizotinib and highlights persistence of the target EML4-ALK on initial chronic crizotinib with a complete remission but then slow relapse on crizotinib without a resistant mutation. The patient enrolled on a phase Ib study of crizotinib plus an HSP90 inhibitor [23] and had a near complete remission, however, a persistent pleural effusion led to withdrawal from the study. Cell and molecular analysis of the pleural fluid was positive for adenocarcinoma but FISH confirmed the loss of EML4-ALK inversion, respectively. Moreover, NGS showed the tumor had acquired several novel gene mutations that may be targetable (Figure 6). The potential therapies suggested include CDK46 inhibitor or immune checkpoint therapy (Table 5).

Case #3 is a patient with CUPS harboring a rare *CCNE1* mutation treated with a CDK4/6 inhibitor on a clinical trial. The patient achieved stable disease for 11 months. In the cell cycle, cyclin E1 complexes with and activates CDK2 driving cells through the G1/S phase and is degraded as cells progress through S phase. Over-expression of CCNE1 has been found in many tumor types and can cause chromosome instability with enhanced proliferation. CCNE1/CDK2 phosphorylates NPAT (nuclear protein mapped to the ATM locus), a transcriptional activator of the cell cycle regulated histone gene expression promoting cell cycle progression in the absence of pRB [24]. In case #3, mutated CCNE1 with a high proliferative index suggests that CDK4/6 inhibition could prevent G1 progression and halt cell cycling. In addition to a CCNE1 mutation, concurrent mutations in *AKT3* and *PTEN* were present, suggesting co-targeting with a PI3K or mTOR inhibitor.

Case #10 is a patient with triple-hit diffuse large B-cell lymphoma (MYC/BCL2/BCL6 translocated). The patient received dose-adjusted R-EPOCH immuno-chemotherapy with rapid progression and was enrolled on a novel-novel investigational trial of a BTK inhibitor + fourth generation IMiD + rituximab. The molecular profile prior to initiating therapy (Table 5) indicated multiple pathway defects including a *TP53* dependent G1-aberrant DNA damage response. Drugs targeting epigenetics (e.g., EZH2 or DNMT3A inhibitor), DNA damage response and aberrant cell cycle may have benefitted this patient.

## 3. Discussion

The frequency and distribution of mutant oncogenes and tumor suppressors have redefined taxonomy for most tumor types. The mutational landscape of cancer is made up of a few mutated genes in a high fraction of tumors (‘mountains’) and most genes are altered at relatively low frequencies (‘hills’) [25]. Precision medicine approaches have been evaluated as novel tailored therapies and current trends emphasize characterizing the mutational repertoire. The questions remain as to what constitutes the ‘driver pathways’ that should be targeted. We focused our efforts on a heterogeneous group of cancer patients with metastasis entering early phase investigational agent trials. These patients have had >3 prior therapies. We provide an analysis of *TP53* mutations present at primary and metastatic sites with other genomic aberrations that may guide rational targeted therapeutics. Tp53 current state-of-the-art precludes testing of *TP53* mutant driven transcriptional programs with selective agent (s), since there is a lack of knowledge of these signaling pathways. Individual patients treated on targeted trials are subject to potential selection bias, however, our analyses highlight examples of decision complexity based on molecular profiling.

The mutational profiling of metastatic sites by NGS identify unique genetic aberrations absent at the primary site. Hence, to fully decipher pathobiology and therapeutic response, clonal and sub-clonal genotypes of individual tumors need characterization. Large-scale NGS projects require integration with functional screens to better develop strategies for novel therapeutic combinations. For functional screening to be useful, key metastatic drivers need to be identified to better define driver pathways that can be optimally targeted in anticipation of drug resistance and tumor evolution [26]. We utilized *TP53* mutations as a critical driver at primary and metastatic sites and hypothesized that mutant p53 protein (s) activate unique signaling pathways within a background of epistasis [27]. Our study demonstrates that the enrichment of *TP53* mutations occur at both primary and metastatic sites. This indicates both truncal and acquired *TP53* mutations are most likely from prior therapies. The premise we surmised was that multiple epistatic mutations can have a combination effect, which differs from those they may elaborate individually. We identified *TP53* mutations that may partner with unique co-mutations in colon and lung cancer. There has been a significant effort to restore wild type p53 function [28] in tumors with mutant *TP53*. Phase 1/II clinical trials are ongoing investigating small molecule inhibitors APR-246 (eprenetapopt binds to p53 at two cysteine residues in the DNA-binding domain and stabilizes mutant p53), PRIMA-1 (p53 reactivation and induction of apoptosis) and MDM2 inhibitor AMG 232 that restores p53 tumor suppression by blocking the MDM2-p53 interaction [29]. These p53 targeted small molecular inhibitors are being developed in both solid and hematologic malignancies. These inhibitors are available for testing in *TP53* mutant cell lines and preclinical models to evaluate combination treatments targeting co-occurring gene mutations identified in our study.

In our analyses, we found co-mutations in the DNA damage repair genes (ATM, BRCA2, PRCKDC, PMS2) that highlight consideration for targeting with PARP inhibitors. Mutation in genes that activate multiple signaling pathways for e.g., mTOR and its inhibition by everolimus is a pharmacologic approach to target mutant *TP53* reported to be activated in breast and pancreatic cancer cell lines. Similarly, histone lysine methyltransferases are known to modulate the methylation status of *TP53* at distinct sites. Mutations in *KMT2C*/*KMT2D* can be targeted with specific methyltransferase small molecule inhibitors that disrupt the WDR5-KMT2 interaction [30]. Some of these core complex mutations along with *TP53* mutations that upregulate the activation of specific cellular pathways can only be investigated by whole genome transcription assays in the backdrop of specific *TP53* mutations. We believe that this is a basis for the transcriptomic studies of primary versus metastasis that could translate into unique ‘pathway’ focused therapies.

The current practice focus has been on mutations considered to be clinically ‘actionable’. Comparative analyses of unmatched and matched primary and metastatic sites have shown depletion versus enrichment of certain oncogenic and tumor suppressor mutations respectively [16]. There appears to be a paucity of universal mutations limited to metastatic sites. However, focusing on distinct non-actionable *TP53* mutations that drive unique signaling pathways may address context of vulnerability and define improved methods for rational therapies. These improved methods of rational combinations may help overcome acquired drug resistance at both primary and metastatic sites. It is prudent to develop transcriptional models to identify signaling pathway drivers. Tumor vulnerabilities differ based on specific *TP53* mutations and tumor type, thus multi-mutant, multi-omics strategies are needed to elucidate cancer protecting activities that can be targeted. The goal of precision medicine in oncology will then move a step closer to the realization of implementing unique clinical trials personalized to each patient’s malignancy. These trials will be key to assess targetable and non-targetable genomic aberrations and provide a handle on moving the needle to enhance survival in patients with metastatic disease.

## 4. Materials and Methods

### 4.1. Patient Population

Patients with advanced solid and hematologic malignancies that were referred to the New Therapeutics Program (*n* = 203) were routinely profiled as a standard of care utilizing commercial next generation sequencing (NGS) platforms. Tumor samples taken from primary and metastatic sites (liver, lung, lymph nodes, pleural fluid, etc.) were formalin-fixed and paraffin-embedded (FFPE). Ten case studies of patients’ pre-/post-targeted treatment biopsies were available for analysis. All patient information was de-identified.

### 4.2. Profiling Platforms

We utilized Precipio 421-NGS (includes FISH) (Precipio Diagnostics, New Haven, CT, USA), Caris-Molecular Intelligence (600-NGS, IHC, FISH/CISH) (Caris Life Sciences, Irving, TX, USA), and Foundation One (300-NGS/CNV) (Foundation Medicine, Cambridge, MA, USA) platforms for NGS analysis. Each commercially available platform cannot be compared to each other as they are propriety encrypted; however, all the actionable genes are represented in each of the platforms. Biopsy samples were annotated by histology, primary and metastatic site, biopsy location, gene mutation, mutation count/gene and *TP53* mutations. We focused on mutations that co-occur with mutant *TP53* type at primary and metastatic sites. NGS molecular profiles from each patient and tumor type were categorized into common and unique mutations.

### 4.3. Mutation Analysis in Primary and Metastatic Tissue Samples

Out of 171 cases, gene mutations from 145 unique patient cases (94 with metastatic tumors and 51 with primary tumor) were analyzed. The mutation frequency and enrichment of mutation was estimated independently in primary and metastatic tumors. Relative frequency was calculated, and binomial exact test was performed to estimate the probability of enrichment of gene mutations in primary or metastatic tumors. For each gene mutation, the odds ratio was calculated as relative frequency of a gene mutation to the reference as average relative frequency of all genes. For all analysis *p* value < 0.05 was considered significant. Data was also stratified for organ specific mutations and similar analysis was performed to estimate frequency and enrichment. All analysis and plots were done utilizing R (v 3.4.3).

### 4.4. Gene Mutation Pathways and Protein Interaction

Cytoscape (http://www.cytoscape.org/) was used for the mapping of gene mutation frequencies and enrichment probability to cancer histology and primary site. We utilized Enrichr [31] to estimate enrichment of Reactome pathways [32] for gene mutations with significant representation across all patients. Signaling pathways were also estimated for primary and metastatic samples from specific primary sites using the enrichKEGG and cluster profiler package from Bioconductor (https://Bioconductor.org). Top genes with mutations that were significant in tissue sites were analyzed for enriched Kegg pathways to find out which cellular pathways are altered by co-occurring gene mutations. Patients enrolled in targeted therapy trials were profiled pre-and/or post-treatment for pathway to ascertain response to therapy. Protein interaction analysis was performed using the String database [33].

### 4.5. Three-Dimensional Mapping

The crystal structure of p53 bound to DNA from Protein Data bank (pdb: 3KZ8) was utilized to map *TP53* mutations [20]. The International Agency for Research on Cancer (IARC—Version R 19) *TP53* database was used to help analyze data on human cancer *TP53* gene variations [21].

## 5. Conclusions

The mutational profiling of primary and metastatic sites of cancer patients participating in early phase therapeutic trials provide an opportunity to identify distinct *TP53* mutations that drive unique signaling pathways, which should guide rational drug combinations to abrogate oncogene addiction and drug resistance, hopefully with minimal toxicity to normal tissue.

## Figures and Tables

**Table 1:** Molecular profiles of advanced cancer patients enrolling in phase I trials.

Primary	Number of Patients	Histology	Number of Mutated Genes	Mutated Gene ID′s
Anus	2	Squamous Cell Carcinoma	27	*APC, ARID2, ASXL1, ATRX, CCND1, CDKN1B, CDKN2A, EPHA5, ERBB2, ERBB4, FANCA, FGF19, FGF4, FLT1, INHBA, MED12, KMT2D, MYCN, NKX2-1, PALB2, PIK3CA, POLE, RARA, RB1, SMARCA4, TERT, TSHR*
Appendix	2	Adenocarcinoma	13	*AR, CDK12, FGFR1, FLT3, GATA6, GNAS, KRAS, KMT2A, KMT2C, NOTCH1, PRKDC, SMAD4, SPEN*
-	1	Mucinous Adenocarcinoma	1	*KRAS*
Bladder	2	Transitional Cell Carcinoma	24	*ATM, CSMD3, EP400, EPHB4, FGFR3, FN1, LTF, MAF, KMT2A, MSH6, MTR, MYH9, NUMA1, PDE4DIP, PDGFB, PDGFRa, PIK3C2B, PIK3CA, PRKAR1A, PTEN, SF3B1, THBS1, TP53, WRN*
Brain	1	Astrocytoma	2	*IDH1, TP53*
-	3	Glioblastoma Multiforme	16	*AKT3, BRAF, EGFR, EGFRvIII, ERBB2, ETV4, FLT3, KRAS, NF1, NOTCH2, PTEN, SMAD2, SMO, TNFAIP3, TRRAP, UBR5*
-	1	Medulloblastoma	10	*BCOR, CARD11, FAM123B, GNAS, LZTR1, KMT2D, NRAS, RARA, SMO, TERT*
-	1	Meningioma	5	*FAT1, FGF19, LRP1B, KMT2D, NF2*
Breast	17	Adenocarcinoma	79	*ABL1, ABL2, AKT1, AR, ARFRP1, ARID1A, ASXL1, ATM, BARD1, BCL2L2, BCL9, BRCA2, CCNE1, CDH1, CDK12, CDKN2A, CHD4, CHEK2, CIC, CREBBP, CSMD3, DAXX, DDR2, DST, EGFR, EPHA3, ERBB4, ESR1, FAM123B, FAT1, FGFR1, FGFR3, FH, FLT1, FLT3, GATA3, GRIN2A, HSP90AB1, Her2/Neu, IDH2, JAK1, JAK2, JAK3, KDM6A, KIT, KRAS, MAP2K4, MAPK8, MST1R, MYCL1, MYST3, NF1, NFKB1, NOTCH1, PARP1, PBRM1, PDGFRA, PDGFRB, PDGFRa, PIK3CA, PIK3R2, PMS2, PRKCI, PTCH1, PTEN, PTPN11, PTPRD, RET, RPS6KA2, RUNX1, SDHA, SGK1, TGM7, TLR4, TNK2, TOP2A, TP53, TPR, TSC1*
CUP	8	Carcinoma	90	*ABL2, ACVR2A, AFF1, AKT2, AKT3, APC, AR, ARFRP1, ARID1A, ARID1B, ASXL1, ATM, ATR, ATRX, AXL, BCORL1, BCR, BRCA2, BTK, CCND2, CCNE1, CDH5, CDK12, CDK8, CDKN1B, CHD2, CHD4, CREBBP, CSMD3, CTNNB1, DNMT3A, EPHA3, EPHB1, ERCC1, EZH2, FANCA, FANCE, FAT1, FGF23, FGF6, FGFR2, GATA2, HGF, IL7R, IRS2, KDM5A, KEAP1, KEL, KRAS, LRP1B, MAP2K2, MAP3K1, MDM4, MET, KMT2C, MRE11A, MSH2, MSH6, MUTYH, MYCL1, MYST3, NOTCH1, NOTCH2, NTRK1, NTRK3, PAK3, PDCD1LG2, PIK3CG, PIK3R2, POLD1, PPP2R1A, PRKDC, PTCH1, PTEN, PTGS2, RALGDS, RANBP2, ROS1, RPTOR, SETD2, SMARCA4, SMARCB1, SNCAIP, STK36, SYK, TCF7L2, TP53, TSC2, ZNF217, ZNF703*
Cervix	1	Adenocarcinoma	5	*APC, PIK3CA, PTEN, RB1, TP53*
-	6	Squamous Cell Carcinoma	45	*ABL2, AKT1, ARID1A, ATM, ATRX, AURKB, BCORL1, CASP8, CCNE1, CDH5, CEBPA, CHEK2, CIC, CYLD, EPHA5, FGF23, FLCN, FLT3, GDNF, IDH1, IGF1R, LRP1B, MAP2K4, MAPK8, MED12, KMT2C, MYC, MYH9, MYST3, NOTCH1, NOTCH2, PALB2, PIK3CA, PIK3CG, PIK3R1, PMS2, PRKDC, RAD51, RANBP2, ROS1, SOCS1, TAL1, TCF7L1, TET2, TOP2A*
Colon	28	Adenocarcinoma	87	*ABL1, AKT1, ALK, APC, ARAF, ARID1A, ARID1B, ASXL1, ATM, BRAF, BRCA2, BTG1, CCND2, CCND3, CDK8, CDKN2A, CSF1R, DDR2, DNMT3A, EGFR, ERBB3, ERCC5, FAM123B, FANCA, FANCC, FANCD2, FAS, FAT1, FBXW7, FGFR1, FGFR2, FLT1, FLT3, FLT4, GATA4, GNA11, GNAS, GPR124, Her2/Neu, JAK2, KDR, KLHL6, KRAS, LRP1B, MAP2K2, MAP2K4, MED12, MET, KMT2A, KMT2D, KMT2C, MYC, NOTCH1, NRAS, NTRK1, NTRK2, NTRK3, PDGFRA, PDGFRB, PIK3CA, PIK3R2, PMS2, POLD1, PPP2R1A, PREX2, PRKDC, PTCH1, PTEN, RB1, RET, RICTOR, ROS1, SMAD2, SMAD3, SMAD4, SPTA1, SRC, TAF1, TGFBR2, TNFAIP3, TOP1, TP53, TSC1, TSC2, VEGFA, XPO1, ZNF703*
Endometrium	6	Adenocarcinoma	24	*ABL1, ABL2, APC, AURKA, AURKB, BRCA1, CYLD, EPHA5, ERBB3, ERCC1, FGFR2, IDH1, JAK3, MAP2K4, NF1, NOTCH1, NRAS, NTRK1, PIK3CA, PTCH1, PTEN, TGM7, TP53, TSC2*
Esophagus	3	Adenocarcinoma	54	*ACVR1B, ASXL1, ATM, ATR, AXIN1, BCL2L1, BRIP1, CARD11, CCND2, CCND3, CCNE1, CDKN2C, CEBPA, CREBBP, CTNNB1, DST, EGFR, EP300, EPHA3, FANCC, FANCL, FH, FLT1, GATA4, GATA6, GLI1, IKZF1, INHBA, JAK1, KDM5C, KEAP1, KRAS, LRP1B, MAGI1, KMT2C, MTOR, MYC, NKX2-1, NTRK3, PMS2, RNF43, RUNX1, RUNX1T1, SLIT2, SMARCA4, SPEN, STAG2, TAF1, TOP1, TP53, TSHR, VEGFA, WT1, XPO1*
Head & Neck	1	Carcinoma	3	*ATM, BRCA2, CDKN2A*
-	1	Mucoepidermoid Carcinoma	11	*CDKN2A, CDKN2B, CJD2, CREBBP, EWSR1, KDM6A, KMT2D, NOTCH1, NOTCH3, SPTA1, TBX3*
-	2	Squamous Cell Carcinoma	8	*BRCA2, CDK4, FLT3, KDR, MET, MSH2, NOTCH2, TP53*
Kidney	1	Clear Cell Adenocarcinoma	3	*BRAF, MEK2, NF1*
-	4	Clear Cell Carcinoma	24	*ACVR1B, ATM, AXL, CD79A, CDK4, EPHA3, EPHA5, FGFR1, FRS2, GLI1, HSP90AA1, KEAP1, MDM2, MSH2, PBRM1, PIK3CA, PMS2, POT1, PRKDC, SETD2, SMARCA4, SPEN, TET2, VHL*
Liver	1	Adenocarcinoma	4	*CDKN2A, Her2/Neu, KRAS, TP53*
-	1	Cholangiocarcinoma	15	*APC, ATR, CDH2, CDKN2A, CSF1R, ERCC1, EZH2, FANCD2, FLT1, IL21R, KIT, NOTCH2, NOTCH4, SRC, XPC*
Lung	20	Adenocarcinoma	85	*ABL1, ALK, APC, ARID1A, ARID1B, ATM, BCL6, BCOR, BRAF, BRCA1, BRCA2, CBL, CCNE1, CDKN2A, CDKN2B, CIC, CSF1R, CSMD3, DICER1, DOT1L, DST, EGFR, EP300, EPHA3, EPHA5, ERBB2, ERBB3, ERRFI1, EXT1, FANCG, FGFR2, FLT1, GNA11, HGF, HRAS, Her2/Neu, IRF2, ITGA10, KDR, KEAP1, KIT, KRAS, LRP1B, MAGI2, MAP2K2, MAP3K1, MAP3K7, MET, MLH1, KMT2D, MSH2, MYC, NF1, NOTCH1, NOTCH2, NOTCH3, NSD1, NTRK1, NTRK3, PARP1, PDGFRB, PIK3CA, PKHD1, PMS2, POLE, PRKDC, PTCH1, PTEN, PTPN11, PTPRT, RAD50, RB1, RET, RPTOR, SETD2, SMARCB1, SMO, SOX9, SRC, TP53, TPR, UBR5, VEGFR2, WT1, ZNF703*
-	1	Neuroendocrine Carcinoma	7	*CDKN2A, CREBBP, CSMD3, DOT1L, MAGI1, PBX1, PRKDC*
-	2	Small Cell Carcinoma	12	*EPHB6, JAK1, KDR, MUTYH, NOTCH1, NOTCH4, NTRK1, PDGFRB, PTCH1, SMARCB1, SYK, TP53*
-	3	Squamous Cell Carcinoma	13	*APC, ATM, BRAF, EGFR, GRIK5, IGF1R, KDR, KRAS, LRP1B, NOTCH1, PIK3CA, PTEN, TP53*
Lymph Node	1	Diffuse Large B-Cell Lymphoma	9	*ABL2, AFF1, DNMT3A, EZH2, PIK3R2, PRKDC, PTGS2, STK36, TP53*
-	1	Mantle Cell Lymphoma	6	*ATM, NOTCH2, PTPRT, RPS6KA2, TP53, TSC2*
Not Specified	1	Adenocarcinoma	1	*PDGFRA*
-	1	Diffuse Large B-Cell Lymphoma	15	*BCL2, CBL, DUSP2, GNA13, HIST1H1D, IGH, KDM4C, MAP3K1, KMT2D, MYC, PIM1, PLCG2, RAD50, RB1, TNGRSF14*
-	2	Melanoma	17	*APC, BRCA2, CCND2, CSF1R, EPHA5, GNA13, IDH1, LRP1B, MAP3K1, KMT2C, MYC, NRAS, PMS2, PRKDC, PTEN, TSC1, TSHR*
-	1	Sarcoma	22	*APC, ARHGAP26, ATRX, C17orf39, CREBBP, ELP2, EP300, FANCG, FANCL, FBXW7, FGF10, GNA13, IL7R, KMT2A, KMT2D, NKX2-1, RB1, RICTOR, SDHB, SMARCA4, TP53, ZNF24*
-	1	Squamous Cell Carcinoma	8	*ALK, ATM, BRAF, BRCA1, JAK2, NOTCH1, NTRK1, TP53*
Ovary	14	Adenocarcinoma	51	*ABL1, ALK, APC, ARAF, ARID1A, ARID1B, ATM, ATRX, AXIN1, BAP1, BARD1, BCR, BLM, BRAF, BRCA1, BRIP1, C11orf30, CCNE1, CDH2, CRKL, CSF1R, CTNNB1, DNMT3A, EP300, ERBB2, ERBB3, ERBB4, FANCD2, FBXW7, FLT4, HRAS, KDM5A, KEAP1, KRAS, LRP1B, MAML2, NOTCH1, NOTCH2, NOTCH4, PALB2, PAX8, PDGFRA, PDGFRB, PMS2, PTEN, RET, SDHA, SLIT2, TAF1, TBX3, TP53*
Pancreas	8	Adenocarcinoma	35	*AKT2, APC, ARID1B, BARD1, BRAF, BRCA1, CDH2, CDK6, CDKN2A, CDKN2B, CTNNB1, ERCC4, FBX27, GNAS, HGF, HNF1A, HRAS, KRAS, MEK2, MET, MUTYH, MYST3, PALB2, PDGFRA, PIK3CA, PIK3R3, PMS1, PMS2, RET, RICTOR, ROS1, SMO, STK11, TP53, TSC1*
Peritoneum	1	Carcinoma	2	*EGFR, TP53*
Pharynx	2	Squamous Cell Carcinoma	8	*CHEK2, EPHA7, KDR, MET, PALB2, RAF1, TP53, TSC1*
Pleura	1	Mesothelioma	6	*BAP1, FOXL2, MYCN, NF2, POLD1, SETD2*
Prostate	5	Adenocarcinoma	49	*ABL1, APC, ARID1A, ARID1B, BCL2L2, BCL6, BRCA2, CDKN1B, CIC, ERBB4, FANCC, FAS, FGF6, FGFR2, FGFR3, FLT1, FLT4, GABRA6, GNAS, IDH2, IRF2, LRP1B, LYN, MAGI2, MAP2K4, MAP3K1, KMT2D, KMT2C, NF1, NIN, NTRK1, PIK3C2B, PIK3CA, POLD1, POLE, PRDM1, PREX2, PTCH1, PTEN, RET, RUNX1T1, SDHD, SETD2, SMAD3, TAF1, TCF7L1, TMPRSS2, TP53, ZNF217*
Rectum	2	Adenocarcinoma	20	*APC, ATM, ATRX, FAT1, FGF23, GPR124, IDH1, KLHL6, KRAS, KMT2D, KMT2C, MYST3, NRAS, PDGFRA, PIK3CA, PRKDC, PTEN, RANBP2, SMAD4, ZNF703*
-	1	Melanoma	6	*DAXX, FANCA, NRAS, PMS2, SUFU, TRRAP*
-	1	Squamous Cell Carcinoma	1	*PIK3CA*
Skin	3	Melanoma	26	*ALK, ARID1A, ATM, ATR, BCL2, BRAF, BRCA2, CARD11, CYLD, DDR2, DNMT3A, FLT1, GNAS, IDH1, INPP4B, MAGI2, KMT2A, KMT2C, NRAS, PDK1, PRKCI, RPTOR, SOX10, SPTA1, TERT, TET2*
-	1	Sarcoma	2	*FGFR1, NOTCH1*
Soft Tissue	1	Sarcoma	23	*CCND2, CD36, CDKN2A, CDKN2B, DOT1L, EP300, FANCA, FANCE, FGFR2, GNA12, KIT, LRP1B, MKI67, KMT2A, KMT2C, PRKDC, RAD21, RUNX1T1, TCF3, TP53, TRAF5, TSC2, WDR90*
Uterus	3	Adenocarcinoma	38	*APC, ATR, BCL11A, CATA6, CBL, CDKN2A, DOT1L, ERBB4, FANCD2, FANCF, FAT1, FBXW7, FGF19, FLT4, FRS2, GATA6, IGF1R, KDM5C, KDM6A, KRAS, LRP1B, MAP2K4, MED12, KMT2C, MST1R, MTOR, PDGFRa, PIK3CA, PMS2, PRDM1, PRKDC, PTCH1, RANBP2, RB1, RICTOR, RPTOR, SRC, TP53*
-	1	Leiomyosarcoma	4	*EPHB6, GID4, TET2, TP53*

Combined patient gene mutation profiles showing genes mutated in at least one patient. Profiles obtained with NGS using Precipio-421-NGS, Caris-Molecular Intelligence, and Foundation One platforms. Total patients: 171.

**Table 2:** Distribution of *TP53* mutations among tumor types.

Primary	Histology	Number of Patients	Number of Patients with mutTP53	Fraction Patients with mutTP53
Breast	Adenocarcinoma	17	7	0.41
Colon	Adenocarcinoma	28	21	0.75
CUP	Carcinoma	8	5	0.63
Endometrium	Adenocarcinoma	6	2	0.33
Lung	Adenocarcinoma	20	16	0.80
Ovary	Adenocarcinoma	14	7	0.50
Pancreas	Adenocarcinoma	8	6	0.75

Fraction of patients with mutated TP53 by primary site and histology.

**Table 3:** Catalog of common and unique TP53 mutations for lung cancer.

Protein Mutation	Exon/Intron	Residue Function	Domain Function	Structural Motif
P47S	4 exon	na	Transactivation	N-terminal Transactivation
P58R	4 exon	na	na	N-term
Q100 *	4 exon	na	na	N-term
K132M	5 exon	Buried	DNA binding	L1/S/H2
C141Y	5 exon	Buried	DNA binding	NDBL/beta-sheets
V143M	5 exon	Buried	DNA binding	NDBL/beta-sheets
Y163N	5 exon	Buried	DNA binding	NDBL/beta-sheets
Y163C	5 exon	Buried	DNA binding	NDBL/beta-sheets
R174W	5 exon	Partially exposed	DNA binding	L2/L3
H179L	5 exon	Zn binding	DNA bindin	L2/L3
R181P	5 exon	Exposed	DNA binding	L2/L3
H193R	6 exon	Buried	DNA binding	L2/L3
R196G	6 exon	Buried	DNA binding	NDBL/beta-sheets
S215I	6 exon	Buried	DNA binding	NDBL/beta-sheets
Y220C	6 exon	Buried	DNA binding	NDBL/beta-sheets
M237I	7 exon	Buried	DNA binding	L2/L3
M237I	7 exon	Buried	DNA binding	L2/L3
M237I	7 exon	Buried	DNA binding	L2/L3
R248W	7 exon	DNA binding	DNA binding	L2/L3
R248W	7 exon	DNA binding	DNA binding	L2/L3
E258G	7 exon	Buried	DNA binding	NDBL/beta-sheets
R273L	8 exon	DNA binding	DNA binding	L1/S/H2
P278A	8 exon	Buried	DNA binding	L1/S/H2
R283P	8 exon	DNA binding	DNA binding	L1/S/H2

IARC *TP53* Database search results for TP53 mutations in lung cancer patients. Most mutations are in exons 4, 5, 6, 7, and 8 located in the DNA binding domain. * represents unknown.

**Table 4:** Catalog of common and unique *TP53* mutations for colon cancer.

Protein Mutation	Exon/Intron	Residue Function	Domain Function	Structural Motif
C141Y	5 exon	Buried	DNA binding	NDBL/beta-sheets
R175H	5 exon	Buried	DNA binding	L2/L3
R213L	6 exon	Buried	DNA binding	NDBL/beta-sheets
I232S	7 exon	Buried	DNA binding	NDBL/beta-sheets
M237K	7 exon	Buried	DNA binding	L2/L3
C238S	7 exon	Zn binding	DNA binding	L2/L3
C238S	7 exon	Zn binding	DNA binding	L2/L3
G244S	7 exon	Exposed	DNA binding	L2/L3
G245S	7 exon	Buried	DNA binding	L2/L3
R248Q	7 exon	DNA binding	DNA binding	L2/L3
R248Q	7 exon	DNA binding	DNA binding	L2/L3
R273C	8 exon	DNA binding	DNA binding	L1/S/H2
R273H	8 exon	DNA binding	DNA binding	L1/S/H2
E286K	8 exon	Partially exposed	DNA binding	L1/S/H2
E286K	8 exon	Partially exposed	DNA binding	L1/S/H2
E286G	8 exon	Partially exposed	DNA binding	L1/S/H2
E286G	8 exon	Partially exposed	DNA binding	L1/S/H2

IARC *TP53* Database search results for *TP53* mutations in colon cancer patients. Most mutations are in exon 5, 7, and 8 located in the DNA binding domain.

**Table 5:** Mutational signatures and pathway analysis of pre- and/or post- targeted therapies individualized in advanced cancer patients entering phase I therapeutic trials.

Index Case	Prior Therapy	Targeted or Immunotherapy	Site of Biopsy	Mutational Signature	Pathway Analysis	Clinical Decisions
64 y F with KRAS WT metastatic rectal adenoCA	FOLFIRI/Avastin; Panitumumab; FOLFOX/Avastin	CDK4/6 Inhibitor	B/L pulmonary metastasis → adenoCA of rectal origin	*NRAS, FLT3, KMT2A, TP53, CDK8, BRCA2, DDR2, EGFR, FLT1, GPR124, KMT2C, PRKDC, SMAD4, SPTA1*	*EGFR, HER2 &* *SMAD2/SMAD3:* *SMAD4* signaling Double-strand break repair	1. NRAS mutation explains lack of response to EGFR therapy 2. FLT3 mutation → Regorafenib 3. PARP inhibitor or CDK8 inhibitor
64 y F with metastatic uterine adenoCA	Carboplatin Taxol Doxil	PI3K inhibitor CDK4/6 Inhibitor	Uterus	*ERBB2, BFBXW7, FLT3, NF1, PIK3CA, PTEN, TSC1, DNMT3A, SMARCB1, TET2, ARID1A, ESR1, MDM4, MSH6, ATRX, FGF3, RAD51*	*HER2, PI3K/AKT* & *PI3K* events in *ERRB4* signaling *PIP3* activates *AKT* signaling DNA Repair Aberrations	1. ERBB2 mutation identified however IHC was negative and HER2 not amplified → deferred monoclonal antibody therapy 2. PI3K is as an active pathway 3. FLT3 mutation → off label Sorafenib recommended 4. Epigenetic therapy with PARP inhibitor or Aurora kinase inhibitor
66 y F with hx of early stage breast cancer develops L supraclavicular LAD biopsy proven -Neuroendocrine Carcinoma, unknown primary	Carboplatin & Etoposide	CDK4/6 Inhibitor	Diffuse LAD	*NTRK3, PTEN, TCF7L2, SMARCA4, AKT3, CCNE1, ERCC1, FANCE*	*FGFR, BCR, PI3K, ERBB2* and *ERBB4* signaling Negative regulation of the *PI3K/AKT* network Active Cell Cycle	1. Due to CCNE1 mutation → CDK4/6 inhibitor trial. Stable disease at C11. 2. PI3K pathway is active suggesting next therapy if patient progresses
69 y F with stage IV (T4N0M1b) NSCLC	Carboplatin & Pemetrexed Anti PD-L1	CKD4/6 inhibitor + anti-VEGFR2	Lung Nodule	*KRAS, TP53, CDKN2A, BRCA2,* *[cMET,* *EGFR, PD-1+,* *PD-LI-] IHC*.	*EGFR, ERBB2 &* FGFR signaling Oncogene induced senescence Immune Checkpoint	1. CDKN2A mutation → CDK4/6 inhibitor trial + anti-VEGFR2 2. Immune checkpoint therapy
52 y F with metastatic EML4-ALK NSCLC	Crizotinib; Crizotinib + HSP90 Inhibitor ChemoRT to the R hilum	Crizotinib	L supraclavicular node	Persistent *ALK* + by IHC & FISH. No *ALK* mutation within *EML4-ALK* translocation; *PD*-1 and *PD-L1* negative, *BRCA2, FGFR1, NOTCH1*	*FGFR* and *FGFR1* ligand binding, activation & signaling Receptor-ligand binding initiates second proteolytic cleavage of NOTCH receptor Double-strand break repair	1. Continue Crizotinib as there is no new mutation acquired in the ALK domain. 2. Investigate FGFR1 mutation as an active driver of potential clinical relevance and laboratory focus.
60 y M with metastatic squamous cell carcinoma of the lung	ChemoRT	Anti-PDL1 antibody	Lung	*PD-1+, APC* *PTCH1, c-MET; TL3, TOPO1; TUBB3*	Beta-catenin phosphorylation cascadeTruncated *APC* mutants & deletions of *AMER1* destabilize the destruction complex	1. AMER1 mutation, a tumor suppressor gene resulting over-activity of the Wnt signaling pathway. 2. Immune checkpoint
61 y F with metastatic lung adenocarcinoma with EGFR exon 19 deletion and HER2 amplification by CISH/IHC	Tarceva Afatinib	Monoclonal antibody to HER2	Lung Pleural fluid	Pre-targeted therapy: *EGFR Exon 19 Deletion (L747_S752 del)* *ERBB2* amplification *(FISH/CISH 6.4)* *PIK3CA* *TOPO2A* *TP53* *KEL* intron 3 Rearrangement Post-targeted therapy: Loss of *ERBB2* amplification by FISH and IHC *EGFR* Exon 19 deletion Loss of *PIK3CA* *FLT3 (V194M)* *TP53* *TOPO2A* *PD-1* negative *PD-L1* negative	*EGFR, ERBB2, FGFR* & *PI3K* Signaling *PI3K/AKT* activation G1/S DNA Damage Checkpoints	1.Tumor evolution across therapy—Loss of target 2. Network analysis reveals alternate activated pathways- PI3K and DNA repair
60 y F with metastatic adenocarcinoma of the lung with EGFR INDEL (exon 19) mutation	Carboplatin Pemetrexed Bevacizumab	Tarceva Afatinib	Lung nodule	Pre-targeted therapy: *EGFR* (*INDEL*) exon 19 *TP53* *CSF-1R* *PMS2* *ARID1A* *PKHD1* *PTPRT* *TPR* Post-targeted therapy: *EGFR (INDEL)* exon19 *EGFR (T790M)* *TP53* *CSF-1R* *PD-1* positive *PD-L1* negative *c-MET* positive *M237I*	p-53 dependent G1/S DNA Damage Checkpoint *EGFR* & *ERBB2* signaling	1. Recommend AZD9291 ± Mab to PD-L1 or Mab to MET 2. Consider MEK inhibitor3. Consider Osimertinib
52 y F with Stage IV EML4-ALK NSCLC	Crizotinib	HSP90 inhibitor + crizotinib	Persistent R pleural effusion→ moderately differentiated adenocarcinoma	Loss of *EML4-ALK* by *FISH, CDKN2A,* *CSMD3, MAGI1, CREBBP, DOT1L, PBX1, PRKDC*	Pre-NOTCH Transcription and Translation Double Strand Break Repair Notch-HLH transcription pathway	1. Loss of ALK (inversion) 2. Alternate activated pathways for future targeting with epigenetic therapy, DNA repair inhibitors and cell cycle inhibitors 3. Anti-PD-1 Mab
10. 74 y F with stage IVA triple hit DLBCL	R-EPOCH X 6 cycles	IMid + BTK inhibitor + Rituximab	Axillary Lymph Node	*TP53* *PIK3R2* *PTGS2* *STK36* *EZH2* *DNMT3A* *PRKDC* *ABL2* *AFF1* *BCL-2* *BCL-6* *c-MYC*	Epigenetic regulation of gene expression Double-strand Break Repair CD28 dependent PI3K/Akt signaling Pre-NOTCH Transcription and Translation TP53 Dependent G1 DNA Damage Response	1. Epigenetic Therapy (e.g., EZH2 or DNMT3A inhibitor) 2. PI3K inhibitor + anti-CD20 Mab 3. STK36 Hedgehog pathway3. CAR-T

## Data Availability

The data presented in this study are available within the article or supplementary material.

## Supplementary Materials

The following are available online at https://www.mdpi.com/2072-6694/13/4/597/s1. Figure S1: The crystal structures of p53 bound to DNA was utilized to map common and rare p53 mutations detected in our cohort of patients. Figure S2: *TP53* protein interaction network from String db.
