# Supplementary material for: TP53 Mutations as a Driver of Metastasis Signaling in Advanced Cancer Patients
Source: Cancers (Basel). 2021 Feb 3;13(4):597. doi: 10.3390/cancers13040597 (PMC7913278; doi:10.3390/cancers13040597)
Supplement: Supplementary file 1 [file cancers-13-00597-s001.pdf]

cancers

MDPI

Supplementary Materials: *TP53* Mutations as a Driver of Metastasis Signaling in Advanced Cancer Patients

Ritu Pandey, Nathan Johnson, Laurence Cooke, Benny Johnson, Yuliang Chen, Manjari Pandey, Jason Chandler and Daruka Mahadevan

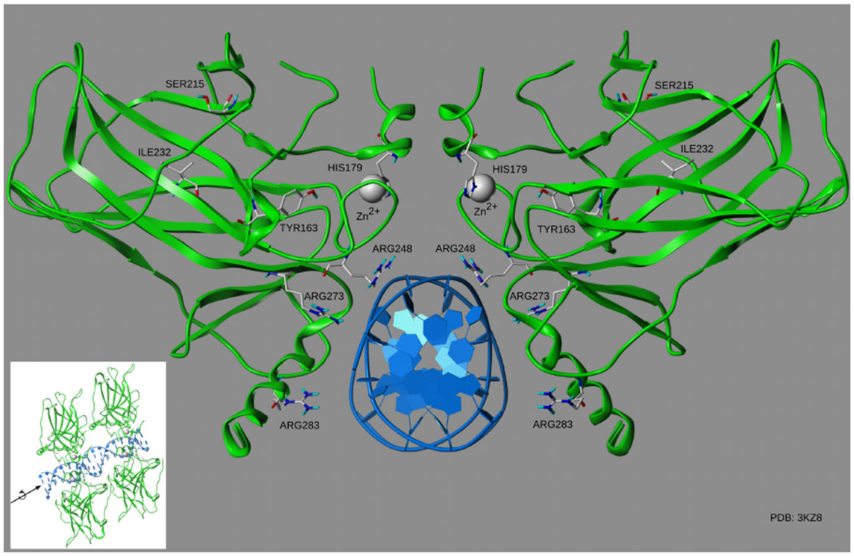

Figure S1. The crystal structures of p53 bound to DNA was utilized to map common and rare p53 mutations detected in our cohort of patients.

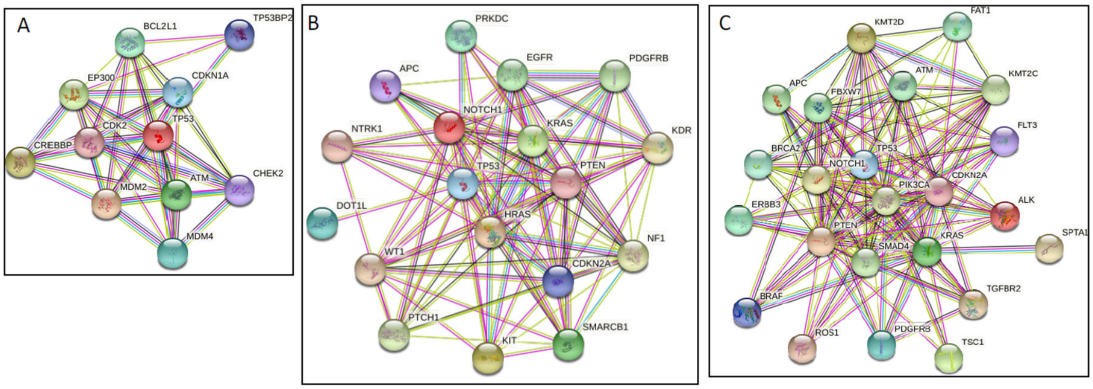

Figure S2. *TP53* protein interaction network from String db. (**A**) Interacting partners of *TP53*. (**B**) Interaction network for top mutated genes in metastatic lung and (**C**) colon adenocarcinoma tissues.
